# Corporate Environmental Performance in China: The Moderating Effects of the Media versus the Approach of Local Governments

**DOI:** 10.3390/ijerph18010150

**Published:** 2020-12-28

**Authors:** Zhiru Guo, Chao Lu

**Affiliations:** 1 China Center for Energy Economics Research, School of Economics, Xiamen University, Xiamen 361005, China; CarrieGuozhiru@163.com; 2 School of Economics and Management, Beijing Jiaotong University, Beijing 100044, China

**Keywords:** media attention, corporate environmental performance, local governments attach importance to environmental protection, property nature

## Abstract

This article selects the listed companies in China’s A-share heavy pollution industry from 2014 to 2018 as samples, uses a random effect model to empirically test the relationship between media attention and corporate environmental performance and examines the impacts of local government environmental protection and property nature on that relationship. Results are as follow: (1) Media attention can significantly affect a company’s environmental performance. The higher the media attention, the greater the company’s supervision and the better its environmental performance. (2) In areas where the government pays less attention to environmental protection, the impact of media on corporate environmental performance is more obvious, but in other areas, the impact of media on environmental performance cannot be reflected; (3) The media attention is very significant for the environmental performance improvement of state-owned enterprises, and it is not obvious in non-state-owned enterprises. (4) A further breakdown of the study found that the role of media attention in corporate environmental performance is only significant in the sample of local governments that have low environmental protection and are state-owned enterprises. This research incorporates the local government’s emphasis on environmental protection into the research field of vision, expands the research scope of media and corporate environmental performance, and also provides new clues and evidence for promoting the active fulfillment of environmental protection responsibilities by companies and local governments.

## 1. Introduction

In recent years, an increasing number of people have suffered from air pollution and the plastic products pollution of the ocean and land has become more severe. The world environmental pollution situation is not optimistic, and environmental governance has become a global problem that needs to be solved urgently. Among the countries that are striving for development with environmental protection, China’s green development process is remarkable: The 18th National Congress of the Communist Party of China put forward the concept of “ecological civilization construction” and formed a “five-in-one” general layout (the term “five in one general layout” refers to the five-in-one integration of economic construction, political construction, cultural construction, social construction and ecological civilization construction, and comprehensive progress), aiming to take into account the ecological governance work such as resource conservation and environmental protection in the development process. In 2014, China promulgated the new “Environmental Protection Law”, which intensifies the punishment of environmental violations, gives the environmental protection department more powers, and require companies to disclose pollutant emission information. It is said to be the most stringent environmental protection law in history; In 2015, “the Overall Plan for Ecological Civilization System Reform” was introduced. This proposes clear and specific action guidelines for ecological civilization construction, so that ecological construction can be based on evidence. All of these formulate a course of action for China’s long-term green development plan from a macro perspective. Considering from the micro level, as the main body of economic activities, the environmental performance of an enterprise is closely related to the environmental protection performance of the entire society. In 2018, start to implement “Environmental Protection Tax Law”. Compared with China’s previous pollution charges set by regulations, this time the levy of pollution taxes in the form of legislation is more formal, and enterprises will also be subject to stricter restrictions when paying taxes. It indirectly reflects that the company’s environmental performance has received the attention of the Chinese government. Alsayegh et al. found that environmental performance and social performance are significantly positively correlated with economic sustainability performance, indicating that the economic value of enterprises and socially created value are interdependent [1], and it also provides a theoretical support for the government to attach importance to corporate environmental performance.

Although there is a certain policy foundation at this phase, as China’s social structure is still in the stage of continuous improvement and the legal system is not yet complete, there are still problems in the implementation of relevant supervisory agencies, and companies that damage the environment, violate social responsibilities and operate illegally are still exist. Under such environmental system conditions, informal systems such as the media are playing an important role in alternative supervision [2]. Wang et al. found that the Environmental Non-Government Organization (ENGO) can alleviate environmental problems by mobilizing local citizens to actively participate in environmental governance [3]. The “melamine” incident (in 2008, many babies who ate milk powder produced by Sanlu Group were found to have kidney stones, and the chemical raw material melamine was subsequently found in their milk powder), the Shuanghui “clenbuterol” incident (in 2011 it was exposed that Henan Jiyuan Shuanghui Food Co., Ltd. had been clenbuterol contaminated pork to produce meat products for many years) and the Wuliangye Group’s interest transfer incident all entered the public eye after they were first reported by the media, causing concern from all walks of life and even the intervention of administrative agencies before they were finally resolved finally resolved. Media supervision has become increasingly effective in the external governance of listed companies nowadays [4]. Zhai et al. proved that media supervision can reduce internal corruption to a certain extent through empirical research [5]; there are also documents that find that media reports can make executive compensation contracts more effective [6]. Some scholars also found in research that negative media reports would reduce the impact of environmental information disclosure quality on reducing the company’s debt financing costs [7]. Zhou et al. found that active media attention will reduce the “range effect” of carbon risk on debt financing costs [8]. Studies have shown that negative media reports have a certain relationship with corporate earnings and stock price trends [9]; but Blitz et al. denied that media attention is the driving factor leading to stock price fluctuations [10]. Dyck et al. found that increased media attention will increase the company’s probability of correcting violations through empirical evidence [11]. At the same time, as an information intermediary, the media also provides more information to the public, reducing the degree of information asymmetry between the public and the enterprise [12]. However, You et al. found that media with different property rights have different reporting quality and tendencies, and different types of media organizations have different effects on corporate governance [13]. Choi et al. found the value of an enterprise increases significantly with the improvement of environmental management or operational performance and believes that the enterprise itself has the motivation to improve environmental management [14].

The impact of media attention on corporate environmental governance has received increasing notice from the academic community nowadays. Li et al. selected listed companies in China’s country’s heavy-polluting industries as samples in their research and found that media supervision can significantly promote enterprises to actively fulfill their social responsibilities [15]; El Ghoul et al. found that this conclusion is more obvious in countries where the media has more freedom [16]. Zhang et al. found that the media’s supervision of public opinion, on the premise of independence and authenticity, will also play a positive role in regulating the environmental management system certification that affects the environmental performance of enterprises [17]. Most scholars believe that media attention can increase the attention of companies, thereby urging companies to actively take measures to solve existing environmental problems and indirectly improve corporate environmental performance [18,19].

At this stage, research on media attention and environmental performance has achieved fruitful results. In China, an emerging transitional country, local governments have direct administrative responsibility for local environmental protection, but existing research has rarely paid attention to the impact of local government protection emphasis on media attention and corporate environmental performance. There is currently some literature on the impact of government regulations on environmental performance. Zheng et al. considered the impact of institutional background on corporate environmental performance in their research [20]; Chen et al. made local environmental regulations as a moderating factor and found that local government environmental regulations have a positive effect on corporate environmental performance [21].

Summary of previous studies at home and abroad found that in recent years, there has been a large number of relevant literature on the role of corporate governance in the media, most scholars have recognized the role of media supervision over listed companies, and in-depth analysis of the way to achieve the role of media supervision and governance, but there are also a few scholars have questioned the role of media supervision, scholars have not yet reached a consensus on the impact of media attention on corporate environmental performance, the results of research have provided the basis for a more comprehensive understanding of the impact of media reporting on corporate environmental governance and long-term development prospects, there are some areas to continue to improve. In the past, when studying institutional factors, the literature often takes the new national macro policy, the degree of administrative intervention, or other institutional environmental factors such as the laws of various regions as the starting point.

In general, there are still different views on whether media governance can have a positive impact on corporate environmental performance, and the role of media corporate governance also needs more multi-dimensional empirical verification. Can the media play the supervisory function of informal system under the role of information intermediary and reputation incentive? How does the local government attach importance to the degree of environmental protection and the nature of enterprise ownership regulate the relationship between the two? Is there a relationship between enterprise environmental performance and its property right? This paper will focus on these problems to carry out empirical research.

Based on it, this article selects 2014–2018 listed companies in China’s A-share heavy pollution industry as a sample, and uses a random effects model to empirically test the relationship between media attention and corporate environmental performance, focusing on the differences between the two in terms of the importance of local government environmental protection and the nature of property rights, and puts forward corresponding policy recommendations based on China’s reality. The main contributions of this paper are: (1) The introduction of the variable of the importance of local governments on environmental protection, exploring its influence on the relationship between media attention and corporate environmental performance, and enriching research in related fields. (2) Combining China’s unique People’s Congress system, this article uses text analysis to measure the importance of environmental protection by the provincial governments by sorting out the proportion of the key words related to environmental protection in the work reports of the provincial people’s congresses in the entire report. (3) The research results of this paper found that the media attention’s effect on the environmental performance of enterprises is only significant in the samples of state-owned enterprises that have low emphasis on environmental protection by local governments. In other words, the media attention has formed an alternative relationship with the local government’s emphasis on environmental protection. At the same time, state-owned enterprises pay more attention to media reports based on the reputation mechanism.

The other parts of this article are arranged as follows: Section 2 is the literature review and hypothesis proposal, Section 3 is the empirical research design, Section 4 is the analysis of the empirical results, and Section 5 is the research conclusions and prospects.

## 2. Literature Review and Hypothesis

### 2.1. Media Attention and Corporate Environmental Performance

Based on media reports that have corporate governance functions, media attention can affect corporate environmental performance. Dyck et al. believe that there are three main mechanisms for the media to achieve corporate governance effects: one is to attract the attention of the government or related institutions, so that administrative forces can intervene; the second is to use the reputation incentive mechanism of executives; the third is to use the power of public opinion [11]. It has been recognized by most scholars.

On the one hand, media reports will arouse widespread public attention and form public opinion. Under market pressure, executives and companies will take the initiative to correct violations and improve governance. In addition, the numerous existing private reporting channels provide the public with a platform for indirect supervision, which will also trigger administrative intervention. Joe et al. found that media exposure can correct wrong decisions, improve the efficiency of the board of directors, and indirectly help increase company value [22]. Li and Shen selected 50 companies with the worst board of directors as a sample and tested the governance role of the media in listed companies through empirical analysis. They found that the media’s exposure to such companies will eventually induce the involvement of relevant administrative agencies to prompt these companies to correct related governance issues [23]. Another study found that media supervision is increasingly effective in the external governance of listed companies, and its mechanism is mainly to cause administrative intervention [4].

The study of Bushee et al. shows that the role of the media has gradually changed from “transmitter” to “commenter” [24]. Media reports with negative sentiment convey more internal company information to shareholders and other stakeholders. Thereby damaging the image of company executives, affecting their future remuneration packages and career prospects, so executives will restrain their behavior and be alert to negative media report. Dyck et al. found that lobbying will significantly increase overseas media’s attention to corporate violations, while increased media attention will raise the company’s probability of correcting violations [11]. They also confirmed that this effect is mainly achieved through two mechanisms: International reputation mechanism and trigger the intervention of supervisory authorities. Luo found that media reports can make executive compensation contracts more effective [6].

In terms of corporate environmental governance, Aerts and Cormier analyzed the content of media reports, and the research verified that there is indeed a correlation between media supervision and corporate environmental communication [25]. Li found that media attention can improve the quality of corporate carbon information disclosure [26]. There are also many documents that prove that media attention will significantly improve the level of corporate environmental governance, including making companies pay more attention to environmental issues, disclose more environmental information, and increase environmental protection investment [17,19,27]. Accordingly, the first hypothesis is proposed:

**Hypothesis** **1 (H1).** *Media attention will significantly improve corporate environmental performance.*

### 2.2. The Impact of Local Government Attention on Media Reports and Corporate Environmental Performance

Since there is an increasing concern to environmental protection among the country, when assess the general performance of local government, environmental performance is also one of the major valuation factors, so the local governments would like to pay more attention to environmental protection. This leads some scholars to find the government’s impact on environmental performance. For example, Chang et al. found that China’s new energy efficiency publicity system can significantly improve the environmental performance of enterprises [28]. Hafezi et al. suggested that the government should adopt appropriate regulatory systems to restrict corporate behavior and avoid situations where environmental performance is sacrificed for profit maximization goals [29]. Research by Zhang et al. showed that during the selection period of the National Civilized City Awards, civilized city enterprises performed better in terms of environmental performance, especially among state-owned enterprises [30]. However, there are also some studies that believe that government regulations have little effect on corporate environmental performance. For example, Graafland et al. found that compared to complying with government supervision, the pressure of social permits perceived by companies can stimulate the improvement of environmental performance better [31]. Some studies have taken policy and institutional factors and regional differences into consideration when examining the role of corporate governance that the media has focused on. Some scholars have made local environmental protection regulations as a moderating factor and found that the environmental protection regulations of local governments have a positive effect on the environmental performance of enterprises [19,21,32,33]. These research results show that although media supervision already has a certain corporate governance function, strong regulatory constraints also have a certain effect on improving the environmental performance of enterprises, and will enhance the governance effect of media.

Some scholars are also concerned about the impact of policy orientation. Bi et al. found that “green finance” policies have certain governance functions on corporate environmental performance. There are also documents starting from the perspective of government intervention to study the impact of this external factor on the relationship between the media and corporate environmental governance [34]. Through empirical research, Wang found that compared with choosing direct intervention strategies, government select indirect intervention methods which is more effectively allowing companies to improve environmental performance and beneficial to optimizing the overall environmental quality of society [35]. But Zhang’s research results are different from common sense. He found that the intensity of government intervention and the level of corporate environmental performance even show a negative correlation through empirical evidence [36]. Liang and Gao found that local officials did not hesitate to collude with some environmentally damaging companies in order to have better economic performance during their term of office, thus greatly destroying the impact of the formal system of the government and other institutions on corporate environmental governance [37]. Based on this, this article combines the National People’s Congress system with Chinese characteristics and comprehensively considers regional differences from a policy perspective to study the impact of the local government’s environmental emphasis on the level of media corporate environmental governance at the provincial people’s congress. This article believes that in areas where the local government attaches great importance to environmental protection, there is a relatively complete environmental protection regulatory system, and corporate environmental performance has certain standards and requirements. In this case, the media as an informal system is very effective. In this case, the media as an informal system has very limited effect. However, in provinces and cities where the local government attaches little importance to environmental protection, corporate environmental protection-related management and implementation systems still need to be perfected. At this time, media reports on corporate environmental protection-related events and news triggers public opinion to promote corporate environmental performances; All in all, this article believes that the media is a substitute. Accordingly, the second hypothesis of this article is proposed:

**Hypothesis** **2 (H2).** *In regions where the local government pays different attention to environmental protection, the effects of media attention on corporate environmental governance varies.*

### 2.3. The Impact of Local Government Attention on Media Reports and Corporate Environmental Performance

Liu et al. believe that state-owned enterprises and non-traditional enterprises have unequal shares in terms of policy benefits and financing preferences [38]. Companies with different nature of ownership will be different in market competition pressure and executive selection and recruitment mechanisms. The role of media attention on corporate supervision is partly achieved by influencing executives and corporate reputation. Under different ownership properties, the corporate governance effects of media may be not the same, and the impact on corporate environmental performance may also vary.

Research by some scholars has confirmed this point. Tang et al. found that social trust can enhance the corporate governance effect of media attention. Compared with state-owned enterprises, this synergy effect is more significant in private enterprises [2]. Li et al. believe that media supervision has a stronger impact on state-owned enterprises that are more strictly regulated by the government [15]. When state-owned enterprises have negative news, the media also tends to cater to readers and report on these state-owned enterprises at the center of public opinion. Through empirical findings, media reports have a stronger effect on urging state-owned enterprises to actively fulfill their social responsibilities than private and other listed companies. Lu et al. carried out a more detailed classification of the nature of enterprises. Their research found that the Internet and policy-oriented media can promote central state-owned enterprises and private listed companies to improve the quality of internal control, but they lose the effect of supervision on local state-owned enterprises [39]. Hu et al. quantified corporate ownership and found that in listed companies with a low ratio of state-owned shares, fulfilling corporate social responsibility has a more significant positive impact on the company’s market value performance [40]. Cheng et al. found that enterprises receiving higher levels of public attention, especially state-owned enterprises, have better environmental performance [41]. Based on the above literature research results, the third hypothesis of this article is proposed:

**Hypothesis** **3 (H3).** *For companies with different ownership properties, the impact of media attention on environmental performance is different.*

**Figure 1:**
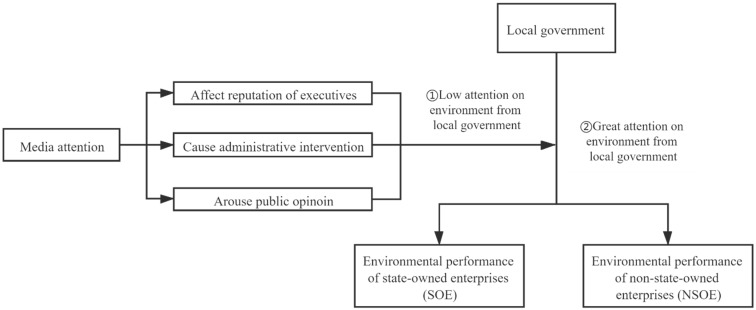
Logic analysis diagram.

The overall logic of this paper is shown in Figure 1 as follows: As an indirect manifestation of local environmental regulations, local governments’ emphasis on environmental performance often imposes certain rigid requirements on corporate environmental performance. This article believes that in areas where the local government attaches great importance to the environment, the environmental governance of enterprises is mainly restricted by local environmental protection policies and systems, and the relationship with the media is relatively weak; While in areas where the local government’s environmental emphasis is low, media attention, as an informal system, has an impact on the environmental performance of state-owned enterprises and non-state-owned enterprises by influencing the reputation of executives, triggering public opinion, and causing administrative intervention. Because state-owned enterprises pay more attention to reputation and are subject to stricter administrative constraints, the impact of media attention on the environmental performance of the two types of enterprises is different.

## 3. Materials and Methods

### 3.1. Sampling and Data Source

This study selects China’s A-share listed companies in heavy pollution industries (the heavy pollution industry sector covers 16 industries, such as thermal power, steel, cement, electrolytic aluminum, coal, metallurgy, chemical industry, petrochemical, building materials, paper making, brewing, pharmaceutical, fermentation, textile, leather making and mining) from 2014 to 2018 as the research sample, and constructs an empirical study of media attention, corporate environmental performance, and local governments attach importance to environmental protection, actual corporate controllers and various control variables. Among them, the local government’s emphasis on environmental protection is measured by using text analysis to sort out the proportion of keywords related to environmental protection in the work reports of the provincial people’s congresses to the total number of words in the report, and this process is achieved by Python. Other data are derived from the China Stock Market & Accounting Research (CSMAR) database.

### 3.2. Variable Selection

#### 3.2.1. Corporate Environmental Performance

This article refers to the practice of Hu [42] and Zhang [17] using the logarithm of the 10,000 yuan unit asset emission fee as an indicator to measure the environmental performance of the company. The pollutant discharge fee of a company is levied based on the total amount of all solid, liquid, gas and other hazardous waste discharged by the company. This indicator can more comprehensively reflect the cost of the company’s treatment of pollutants. This indicator is a negative indicator that means the higher the corporate pollution fee, the worse the corporate environmental performance.

#### 3.2.2. Media Attention

The existing literature has roughly the same measurement methods for media reports. They measure media attention by counting the number of media reports on companies. This article will refer to the practice of Wang et al. [19] and use the CSMAR news media database as the source of media data. After the environmental protection keywords (pollution, destruction, sewage discharge, secret discharge, waste water, exceeding standard, leakage, explosion, death, accident, safety, violation, smoke and dust, oil spill, oil spill, dam break, loss, gas, carcinogenic, poison, blacklist, deforestation, illegal, investigation, waste gas, waste residue, blacklist, evil, dirty, rectification, rectification, sewage, black smoke, mycin, odor, noise, waste, dumping, chromium slag, slag, manganese slag, poisonous gas, mud, blood lead, waste dust, black powder, radioactivity, harmful, excessive, environmental violation, environmental damage, pollution reduction, environmental protection, environmental protection, green, ecology, energy saving, emission reduction, green water, green mountains, clean, environmental protection, tree planting, afforestation) are screened, the original data of the annual report is obtained, and the processing of ln (1+media report volume) is adopted.

#### 3.2.3. Local Governments Attach Importance to Environmental Protection

This article combines China’s unique People’s Congress system and uses Python programming to calculate the proportion of the frequency of environmental protection keywords (reduce pollution, protect the environment, environmental protection, green, ecology, energy saving, emission reduction, green water, green hills, clean, environmental protection, tree planting, afforestation) in the total number of characters in the work reports of the provincial people’s congresses, and uses one hundred times that ratio to measure the degree of local government’s attention to environmental protection. Comparing the importance of environmental protection by local governments with the national average for the year, the provinces that are higher than the national average are classified as areas with high government environmental value (Gov_H). The regions below the national average are classified as areas where the government pays less attention to environmental protection (Gov_L).

#### 3.2.4. Enterprise Nature

According to the nature of the actual controller of the listed company, the company is divided into state-owned enterprises (SOE) and non-state-owned enterprises (NSOE), of which non-state-owned enterprises mainly include private enterprises and foreign-funded enterprises.

#### 3.2.5. Control Variables

Drawing on the previous literature [2,19,42], the control variables of this article are selected as shown in the Table 1 follows: operating efficiency, company years, proportion of independent directors, proportion of the company’s largest shareholder and whether to disclose social responsibility annual report.

### 3.3. Construction of Models

Based on the selection of the above key indicators, construct a multiple regression model for empirical testing. According to Li et al.’s research, it is found that the lagging media reports have the most obvious impact on the fulfillment of corporate social responsibility [15]. Establish the model (1) to study the impact of media attention and reports on corporate environmental performance, and test Hypothesis 1:

CEP_t_ = α_0_ + α_1_ Media_t−1_ + α_2_ CE_t_ + α_3_ Ind_t_ + α_4_ BSR_t_ + α_5_ Age_t_ + α_6_ Disclosure_t_ (1)

Then calculate the annual average according to the percentage of the frequency of environmental protection keywords reported by the provincial and municipal governments in the total number of characters. According to the relationship between the importance of environmental protection by local governments in each province and the average value, the enterprises are divided into two groups with high and low attention on environmental protection by the local government. The study respectively substituted two sets of data into the model (1) to study the differences in the impact of different local governments’ environmental protection attention on the impact of media attention on the environmental performance of enterprises to verify hypothesis 2. Finally, according to the nature of the actual controller of each listed company, it is divided into state-owned enterprises and non-state-owned enterprises, and the data is substituted into model (1) to study the influence of the nature of property rights on the relationship between media attention and corporate environmental performance, and verify whether hypothesis 3 is true.

CEP_t_ represents the company’s environmental performance in year t; Media_t−1_ represents media attention in the t − 1 year; CE_t_ means business efficiency; Ind_t_ represents the ratio of independent directors in year t; BSR_t_ represents the shareholding ratio of the largest shareholder of a listed company; Age_t_ represents the company’s listing years; Disclosure_t_ represents whether to disclose the corporate social responsibility annual report. Disclosure is 1 and undisclosed is 0. If the hypothesis is established, that is, media attention can indeed have a positive effect on the environmental performance of a company. Considering that the amount of pollution discharge fees and the environmental performance of the company are negatively correlated, the α_1_ in the model should be negative; If assumptions two and three are true, the regression result is significant and the α_1_ in the model is also negative, and the regression results of the two sets of sample data should be different.

In this paper, StataSE 16.0 (StataCorp, College Station, TX, USA) is used to calculate the model. The Hausman test is performed on the model constructed above, and the *p* value obtained from the test is far greater than 5%, and the null hypothesis is not rejected. Therefore, this paper selects a random effects model for empirical testing.

## 4. Empirical Results

### 4.1. Descriptive Statistics

In order to count the basic descriptive characteristics of each variable, first perform descriptive statistical analysis. Table 2 shows the descriptive statistical results of the data. It can be seen from the results: (1) The minimum value of the pollution discharge fee (CEP) is only 49 yuan, the maximum value exceeds 150 million yuan, and the standard deviation (SD) is 2,883,137.9014, indicating that there is a huge difference in pollution discharge fees between sample enterprises, and there are significant differences in environmental governance performance between companies; (2) The maximum and minimum values of media attention data (Media) in the first media are very different, it indicates that the number of media reported by different companies is quite different; (3) Whether to disclose the corporate social responsibility report (Disclosure), the median of the dichotomous variable is 0 and the average is less than 0.5, indicating that less than half of the sample companies that publish the social responsibility report, the corporate social responsibility information disclosure mechanism needs to be strengthened, and the distribution of other variables is within a reasonable range.

Table 3 shows the descriptive statistical results of media attention data in different years. It can be seen that during the period from 2014 to 2018, the average level of media coverage generally showed a trend of first decline and then rise, and the standard deviation (SD) data also showed a decline first and then rise, which shows that Chinese media organizations have different trends in the number of environmental news reports of different companies.

Table 4 shows the descriptive statistical results of the data on the importance of environmental protection by local governments. The companies selected in this paper are distributed in 28 provinces, municipalities and autonomous regions in China. It can be seen that the average level of environmental protection attention of various provincial governments has changed little during the period 2014–2018. But the annual maximum value is almost three times the minimum value, indicating that there are still large differences in the importance of environmental protection by local governments in various provinces at this stage.

### 4.2. Correlation Test

In order to preliminarily judge whether there is a large correlation between the variables, the Pearson test was performed on each variable, and the results are shown in Table 5: (1) The correlation coefficients between media attention (Media) and corporate performance (CEP) are all negative, which can preliminarily reflect that media attention promotes environmental performance. (2) The correlation coefficient between the main variables is less than 0.2, and there is no obvious correlation. The possibility of multicollinearity between the variables can basically be ruled out. This result laid the foundation for the later model construction.

### 4.3. Analysis of Regression Results

#### 4.3.1. Media Attention and Corporate Environmental Performance

Since there is a negative correlation between the 10,000 yuan asset pollution discharge fee and the corporate environmental performance, the less the pollution discharge fee the better the environmental performance of the company, and there is a negative correlation between media attention and corporate performance (CEP). Column (1) of Table 6 shows the unilabiate regression results of media attention and corporate environmental performance. The coefficient of media attention is −0.4704, and it is significant at the 5% level, which preliminarily shows that media attention does have a certain effect on corporate environmental performance. The regression results of adding the control variables are shown in column (2) of Table 6. The coefficient of media attention is −0.4631, and it is significant at the 5% level, indicating that media attention can positively affect the environmental performance of enterprises, hypothesis one is valid.

In addition, observing the control variables found that there is a negative correlation between operating efficiency (CE) and corporate environmental performance, that is, the higher the corporate operating efficiency, the worse the corporate environmental performance, and suggesting some companies may sacrifice environmental performance in order to maximize business profits. There is a positive relationship between the company’s listing age (Age) and environmental performance, indicating that companies that have developed for a certain number of years will pay more attention to the benefits of environmental protection and other social responsibilities under the condition that they have been able to obtain more stable profits. Good environmental performance can establish a more active and responsible corporate image.

#### 4.3.2. The Impact of Local Government’s Emphasis on Environmental Protection on the Relationship between Media Attention and Corporate Environmental Performance

Table 6 (3) and (4) are the regression results of media attention and corporate environmental performance in areas where the government’s environmental emphasis is low and the government’s environmental emphasis is high. The results of (3) in Table 6 show that in areas where local governments pay less attention to environmental protection, the regression coefficient between media attention and corporate environmental performance is −0.6333, which is significant at 1%. It shows that the negative correlation between media attention and corporate environmental performance is significantly established. The results of (4) in Table 6 show that in areas where the local government attaches great importance to environmental protection, media attention has no significant impact on the environmental performance of enterprises. Therefore, the second hypothesis of this article has been verified. That is, in areas where local governments pay less attention to environmental protection, media attention has an increased impact on the environmental performance of enterprises, but in areas where local governments pay more attention to environmental protection, media attention to the environment of enterprises performance has no impact. This result shows that as an informal system, media attention plays an alternative role to government regulations in affecting corporate environmental performance.

#### 4.3.3. Differences in the Impact of Media Attention on the Environmental Performance of Enterprises under Different Ownership

Table 6 columns (5) and (6) are the regression results of media attention and corporate environmental performance under different corporate property rights. The column (5) is the result of the state-owned enterprise group, and the column (6) is the result of the non-state-owned enterprise group. The results of (5) in Table 6 show that the regression coefficient between media attention and corporate environmental performance is −0.7487, and it is significant at 1%, indicating that the positive influence of media on corporate environmental performance in the state-owned enterprise group is significantly established. The results of (6) in Table 6 show that the influence of media attention on the environmental performance of non-state-owned enterprises is not valid. Therefore, the third hypothesis of this article has been verified, that is, the impact of media attention on corporate environmental performance varies significantly among companies with different ownership properties.

#### 4.3.4. Analysis of Further Detailed Inspection Results

Based on the above research results, this paper further divides the sample into four groups in order to perform a more detailed analysis: (1) Gov_H*SOE represents state-owned enterprises that place high emphasis on environmental protection by local governments; (2) Gov_H*NSOE stands for non-state-owned enterprises that place high emphasis on environmental protection by local governments; (3) Gov_L*SOE means state-owned enterprises that place low emphasis on environmental protection by local governments; (4) Gov_L*NSOE is non-state-owned enterprises that place low emphasis on environmental protection by local governments. The corresponding regression results shows in Table 7.

According to the results in columns (1), (2), and (4) of Table 7, in the sample groups of state-owned enterprises that place high emphasis on environmental protection by local governments (Gov_H*SOE), non-state-owned enterprises that place high emphasis on environmental protection by local governments (Gov_H*NSOE) and non-state-owned enterprises that place low emphasis on environmental protection by local governments (Gov_L*NSOE), the media’s attention to corporate environmental governance is not clearly reflected, since there the regression results of the two are not statistically significant.

Observing the regression results in column (3), in the sample of state-owned enterprises with low environmental protection attention by local governments (Gov_L*SOE), the regression coefficient of media attention and environmental performance is −1.1265, and it is significant at 1% confidence level, indicating media attention acts a significant role in promoting environmental performance of those enterprises. The importance of local government environmental protection and the nature of state owned property rights can jointly promote the impact of media attention on environmental performance. These corporate locates in the area where government attaches little importance to environment, then the media acts a substitution effect for local government, so the relationship between media attention and CEP is more significant. In addition, state-owned corporate is more sensitive to be reported by medias, so they prefer to perform better once being exposed by medias, this will also let the media has a stronger supervision effect on this kind of corporate.

Besides, in this column, the regression coefficient of disclosure and environmental performance is −0.6307, with significant level of 1%, which shows there is a significant positive correlation between disclosing the corporate social responsibility annual report and corporate environmental performance, that is, among state-owned companies with low environmental protection attention by local governments, those who disclose the corporate social responsibility annual report performs obviously greater in environmental protection than those who don’t.

### 4.4. Endogenous Test

In terms of this article, there may be the possibility of mutual cause and effect between media attention and corporate environmental performance. Media attention can have an impact on corporate environmental performance, but the increase in media attention may also be due to poor or excellent corporate environmental performance. It is very necessary to perform endogenous test. This article draws on the practice of Wang et al. (2017) and believes that there is a significant influence relationship between media attention and the average number of media reports on the industry (Media_ind). However, the average number of industry reports will only affect the company’s probability of being reported, and has no direct relationship with the company’s environmental performance. Therefore, this article selects the industry average number of reports as an instrumental variable, and uses Stata’s two-stage least squares method to test hypothesis 1. The specific results are shown in the Table 8 as follows:

In the first stage (First-stage), Media is the explained variable, Media_ind is the explanatory variable, the regression coefficient is 0.6128, and the result is significant at 1%, indicating that the higher the industry average media coverage, the higher the media coverage. In the results of the second stage, the fitted value of Media is an explanatory variable with a coefficient of −3.7822, and it is significant at the 1% level, that is, media reports can improve the environmental performance of the company. This result is consistent with the results obtained in Hypothesis 1, and indicating the model is considered to have passed the endogenous test.

### 4.5. Robustness Test

In order to further verify the reliability of the empirical results, this article conducts a robustness test from three aspects: replacing explanatory variables, measuring methods of explained variables, and changing the sample interval.

First, replace the main explanatory variable media attention. This article refers to the practice of Du and Han selects professional media [43]. The current classification standards for professional media have not yet reached a unified standard. According to the content of media reports, this article selects the media of the Ministry of Ecology and Environment and related agencies of various industries as the source of professional media samples (*China Energy News, China Natural Resources News, China Metallurgical News, China Industry News, China Chemical Industry News, China Petroleum News, China Coal News, China Environment News, China Green Times, China Textile News, China Nonferrous Metals News, China Building Materials News, China Petrochemical News, China Mining News, World Metal Herald, and China Coal Geological News*) organizes annual report volume data and takes ln (1 + media report volume) to measure professional media attention. The results in the first column of Table 9 show that the regression coefficient between media attention measured by paper-based professional journals and environmental performance is −0.1520, and it is significant at the 10% level, which confirms that professional media attention has a significant role in promoting corporate environmental performance.

Second, replace the environmental performance of the explained variable. This article refers to the practice of Zhang et al. [44] which selects the total environmental protection renovation expenditure (unit: million yuan) in the RKS database to measure the environmental performance of the company. By consulting the RKS rating system, it can be seen that the environmental score is statistically judged from four dimensions: overall environmental management information, pollution prevention information, sustainable resource use information, and climate change mitigation and adaptation information. The indicator of total expenditure on environmental protection renovation can comprehensively quantify the positive contribution of enterprises to environmental protection, so this indicator is selected to test the robustness of Hypothesis 1. From the regression results in the second column of Table 9, it can be seen that the regression coefficient between media attention and corporate performance measured by total environmental protection renovation expenditure (EI) is 0.3140, and it is significant at the 1% level, which confirms that media attention has a significant impact on improving corporate environmental performance.

Finally, change the sample interval. This article refers to the practice of Liu et al. [45]. Since the 2014 “New Environmental Protection Law” has just been promulgated and corporate environmental governance is in a transitional period, one year of data is reduced and the sample interval is shortened to 2015–2018, and a multiple regression analysis is performed on Hypothesis 1 again. From the regression results in the third column of Table 9, it can be seen that the regression coefficient between media attention and corporate performance is −0.5726, and it is significant at the 5% level, which also confirms that media attention has a significant role in promoting corporate environmental performance.

In summary, by replacing the main explanatory variables, explained variables and changing the sample interval for robustness testing, the results obtained are consistent with the previous empirical results, which proves the reliability of the conclusions in this paper.

## 5. Conclusions and Policy Implications

This article selects the listed companies in China’s A-share heavy pollution industry from 2014 to 2018 as samples, and uses a random effect model to empirically test the relationship between media attention and corporate environmental performance, and analyse the impacts of local government environmental protection and property nature on that relationship. Results are as follow: (1) Media attention can significantly affect a company’s environmental performance. The higher the media attention, the greater the company’s supervision and the better its environmental performance. Combining the theoretical basis of the previous literature, this paper also confirms that media attention has the effect of affecting corporate environmental performance through empirical methods, and further enriches the research on the role of media in corporate governance. (2) In areas where the government pays less attention to environmental protection, the impact of media on corporate environmental performance is more obvious, but in other areas, the impact of media on environmental performance cannot be reflected. It indirectly shows that the media has played an alternative role to government regulation. (3) A grouping study on the nature of corporate property rights found that media attention has a significant effect on the environmental performance of state-owned enterprises, but the relationship between the two does not hold in the non-state-owned enterprise group. State-owned enterprises, while aiming for profit, are also shouldering more social responsibilities such as solving employment and supporting emerging industries and tend to pay more attention to their corporate image. In addition, most state-owned enterprises are directly under the management of relevant state agencies. Therefore, when state-owned enterprises are exposed by the media, executives will actively take measures to correct wrongdoings in a timely manner in order to maintain the corporate image; however, state-owned enterprises are mainly considering profit maximization, especially some newly listed companies, which may be in a state of making ends meet. They may want to attach importance to improving environmental performance without capital strength, and they can only ignore media reports. (4) Further subdivision research found that the effect of media attention on corporate environmental performance was only significant in the samples of state-owned enterprises with low environmental protection attention from local governments, indicating that the high environmental protection attention of local governments and the nature of state-owned property rights can jointly promote media attention’s impact on environmental performance. The research in this article takes the importance of local government environmental protection into the research field of vision, expands the research scope of media and corporate environmental performance, and also provides new clues and basis for promoting enterprises and local governments to actively fulfill their environmental responsibilities.

Combining China’s existing social system and environmental protection policies, the following suggestions are made: (1) Actively use the media’s publicity function. This research confirms that the media has a significant effect on corporate environmental performance, but actually the media does not have enough motivation to supervise each company. If the media can provide the public with a way to report environmentally polluting companies, it will reduce the media’s cost of collecting news, and it also provides a convenient solution for citizens affected by environmental pollution. Besides, local authorities can collaborate with the media to publicize the importance of environmental protection, raise public awareness of environmental protection, and fundamentally improve environmental problems. (2) Establish a relatively complete environmental protection regulatory system. As an informal system, media attention only serves as a substitute for government regulation. The research conclusions also show that the media’s attention to environmental governance has certain limitations, but improving corporate environmental performance is a universal issue that needs to be resolved urgently. If environmental performance of all companies should be affected, it is necessary to establish and improve Chinese environmental protection legal system as soon as possible. Local governments should formulate practical environmental protection rules and regulations based on actual local ecological conditions and regional customs. In addition, when dealing with environmental issues, it is necessary to ensure the enforcement of the law to avoid repeated penalties due to light fines and other penalties. For companies that perform well in the environment, there should also be corresponding incentives to establish a positive atmosphere for environmental protection among companies.

This article also has some shortcomings. Chinese companies have less disclosure of environmental protection related information, and the disclosure format has not formed a unified standard, and many companies have not listed in detail the number of sewage charges. Due to the lack of data, there are many loss samples, which may affect the regression effect. For future research, the environmental protection tax that China has implemented since 2018 is a good indicator of environmental performance by determining the tax amount based on statistics of corporate solid, liquid, and gas pollutant emissions and noise. Besides, due to differences in policy preferences and anti-risk capabilities between SMEs and sci-tech innovation board companies and other companies, there are also differences in production links and business models between companies in different sectors. Future research can further study the differences in corporate environmental performance under various characteristics.

## Figures and Tables

**Table 1:** Variable definition table.

Variable Type	Variable Name	Variable Symbol	Variable Definitions
Explained variable	Corporate environmental performance	CEP	Measured with the natural logarithm of the company’s annual 10,000-yuan asset pollution discharge fee
Explanatory variables	media attention	Media	The number of media reports of the company in the previous year is measured by Ln (1 + number of reports)
Control variables	Operating efficiency	CE	Measured by dividing the company’s current operating costs by operating income
	Independent director ratio	Ind	The number of independent directors in the company’s board of directors accounts for the proportion of the total number of board members
	Proportion of the largest shareholder	BSR	The shareholding ratio of the largest shareholder of the company
	Company years	Age	Measured by Ln (1 + the number of years the company has been listed)
	Information disclosure	Disclosure	Disclosure of social responsibility report as 1, non-disclosure as 0

**Table 2:** Descriptive statistics of variables.

Variables	*n*	Mean	Max	Min	Median	S.D.
CEP	606	990.5628	15,746.4657	0.0049	305.4898	2,883,137.9014
Media	606	0.2607	12.0000	0.0000	0.0000	0.7749
CE	606	0.7910	1.1081	0.1764	0.8020	0.0175
IND	606	0.3688	0.6000	0.2500	0.3333	0.0028
BSR	606	0.3779	0.8999	0.0908	0.3755	0.0241
Disclosure	606	0.3548	1.0000	0.0000	0.0000	0.2293
Age	606	1.3884	2.5808	0.0000	1.4973	0.3946

**Table 3:** Descriptive statistics on the number of media reports.

Year	*n*	Mean	Max	Min	Median	S.D.
2014	121	0.2667	6.0000	0.0000	0.0000	0.7390
2015	125	0.2400	5.0000	0.0000	0.0000	0.7556
2016	122	0.1885	3.0000	0.0000	0.0000	0.5505
2017	132	0.1818	7.0000	0.0000	0.0000	0.7797
2018	106	0.4623	12.0000	0.0000	0.0000	1.4154

**Table 4:** Descriptive statistics on the importance of local governments on environmental protection.

Year	*n*	Mean	Max	Min	Median	S.D.
2014	28	0.7377	1.3433	0.3675	0.7339	0.1860
2015	28	0.7010	1.0491	0.3183	0.7086	0.1967
2016	28	0.7601	1.4155	0.3413	0.7609	0.2228
2017	28	0.7367	1.2631	0.4118	0.7116	0.2138
2018	28	0.8044	1.3230	0.4624	0.8038	0.2189

**Table 5:** Correlation test.

Variables	CEP	Media	CE	Ind	BSR	Age	Disclosure
CEP	1.0000						
Media	−0.0872 **	1.0000					
CE	0.0682 *	0.0348	1.0000				
Ind	−0.0497	0.0102	0.0964 **	1.0000			
BSR	0.0451	0.0211	0.1114 ***	−0.0142	1.0000		
Age	−0.0940 **	−0.0203	0.0591 ***	−0.1138 ***	0.0371	1.0000	
Disclosure	0.0165	0.0150	0.1286	0.0385	0.0942	0.0137 **	1.0000

Note: The data in the table are the regression coefficients and t-values of each variable ***, ** and * are significant at 1%, 5% and 10%, respectively, the same below.

**Table 6:** Media attention and the return of corporate environmental performance.

Variables	(1)	(2)	(3)	(4)	(5)	(6)
Media	−0.4704 **	−0.4631 **	−0.6333 ***	−0.1993	−0.7487 ***	0.1086
	(−2.31)	(−2.29)	(−2.71)	(−0.55)	(−3.03)	(−0.32)
CE		1.0732 *	1.6580 **	0.4352	2.0927 ***	−0.3963
		(−1.92)	(2.55)	(0.43)	(−2.63)	(−0.49)
Ind		2.203 ***	−1.1876	−3.9769 *	−5.3078 ***	−0.2315
		(−3.14)	(−0.71)	(−1.67)	(−2.63)	(−0.12)
BSR		0.4717	0.7174	0.0367	−0.1640	3.1396 ***
		(1.00)	(1.26)	(0.05)	(−0.29)	(−3.79)
Age		−0.3153 ***	−0.3116 **	−0.2904	−0.0230	−0.6641 ***
		(−2.70)	(−2.24)	(−1.45)	(−0.12)	(−2.66)
Disclosure		0.0303	−0.2588	0.2799	−0.2195	0.6838 ***
		(−0.2)	(−1.34)	(1.13)	(−1.17)	(2.66)
Year	No	No	No	No	No	No
Industry	No	No	No	No	No	No
Cons	1.3601 ***	1.6083 **	0.6200	2.8639 **	1.7369 *	1.1484
	(17.32)	(−2.35)	(0.74)	(2.49)	(−1.79)	(−1.18)
Adj. R^2^	0.0094	0.0194	0.042	−0.0016	0.039	0.102
F-statistic	5.34 **	3.00 ***	3.49 ***	0.93	3.54 ***	5.37 ***
*n*	606	606	342	264	374	232

Note: The data in the table are the regression coefficients and t-values of each variable ***, ** and * are significant at 1%, 5% and 10%, respectively, the same below.

**Table 7:** Local governments’ emphasis on environmental protection and the breakdown of property rights.

Variables	Gov_H*SOE	Gov_H*NSOE	Gov_L*SOE	Gov_L*NSOE
Media	−0.0845	−0.2049	−1.1265 ***	0.4152
	(−0.18)	(−0.35)	(−4.13)	(−0.99)
CE	1.9875	−1.0690	2.5460 ***	−0.0570
	(−1.39)	(−0.71)	(−2.79)	(−0.06)
Ind	−8.6800 **	0.412	−3.0405	−1.2602
	(−2.41)	(−0.13)	(−1.31)	(−0.53)
BSR	0.067	2.182	−0.3553	3.3795 ***
	(−0.07)	(−1.45)	(−0.53)	(−3.37)
Age	0.3075	−0.5542 *	−0.2659	−0.7647 ***
	(−0.89)	(−1.78)	(−1.28)	(−3.33)
Disclosure	0.2996	0.4884	−0.6307 ***	0.7377 **
	(0.93)	(1.22)	(−2.81)	(2.17)
Year	No	No	No	No
Industry	No	No	No	No
Cons	2.1368	1.9767	1.1641	1.0680
	(1.27)	(1.19)	(1.02)	(0.89)
Adj. R^2^	0.0147	0.0024	0.1051	0.1496
F-statistic	1.39	1.04	5.19 ***	4.7 ***
*n*	159	105	215	127

Note: The data in the table are the regression coefficients and t-values of each variable ***, ** and * are significant at 1%, 5% and 10%, respectively, the same below.

**Table 8:** Endogenous test results.

Variables	First-Stage	Second-Stage
	Media	CEP
Media		−3.7822 ***
		(−5.05)
Media_ind	0.6128 ***	
	(8.34)	
CE	−0.0525	1.3668 **
	(−0.48)	(2.03)
Ind	0.1415	−2.2438
	(0.53)	(−1.34)
BSR	0.0862	0.6059
	(0.95)	(1.07)
Age	−0.0071	−0.3580 **
	(−0.32)	(−2.56)
Disclosure	0.0085	0.05349
	(0.29)	(0.29)
Year	No	No
Industry	No	No
Con	−0.0289	1.7922 **
	(−0.22)	(2.18)
Adj. R^2^	0.097	−0.4231
F-statistic	11.83 ***	
Wald chi2		34.33 ***
*n*	606	606

Note: The data in the table are the regression coefficients and t-values of each variable ***, ** and * are significant at 1%, 5% and 10%, respectively, the same below.

**Table 9:** Robustness test results.

	Replace Media Attention	Replace Environmental Performance	Narrow the Sample Interval
Media	−0.152 *	0.3140 ***	−0.5726 **
	(−1.68)	(−3.16)	(−2.41)
CE	1.1381 **	−0.5926	1.2353 *
	(−2.02)	(−1.33)	(−1.85)
Ind	−2.4005 *	1.8863 **	−1.9656
	(−1.72)	(−1.86)	(−1.21)
BSR	0.48233	0.8049 **	0.456
	(−1.02)	(−2.32)	(−0.80)
Age	−0.3015 ***	0.2323 **	−0.3694 ***
	(−2.58)	(−2.15)	(−2.73)
Disclosure	0.0634	—	0.1322
	(−0.41)		(−0.73)
Year	No	No	No
Industry	No	No	No
Cons	1.5483	1.4999 **	1.3143
	(−2.26)	(−2.14)	(−1.64)
Adj. R^2^	0.0155	0.0786	0.0234
F-statistic	2.58 **	5.2 ***	2.93 ***
*n*	606	250	485

Note: The data in the table are the regression coefficients and t-values of each variable ***, ** and * are significant at 1%, 5% and 10%, respectively, the same below.
